# High Refractive Index Polymer Thin Films by Charge-Transfer Complexation

**DOI:** 10.1021/acs.macromol.2c02532

**Published:** 2023-03-03

**Authors:** Ni Huo, Wyatt E. Tenhaeff

**Affiliations:** Department of Chemical Engineering, University of Rochester, Rochester, New York 14627, United States

## Abstract

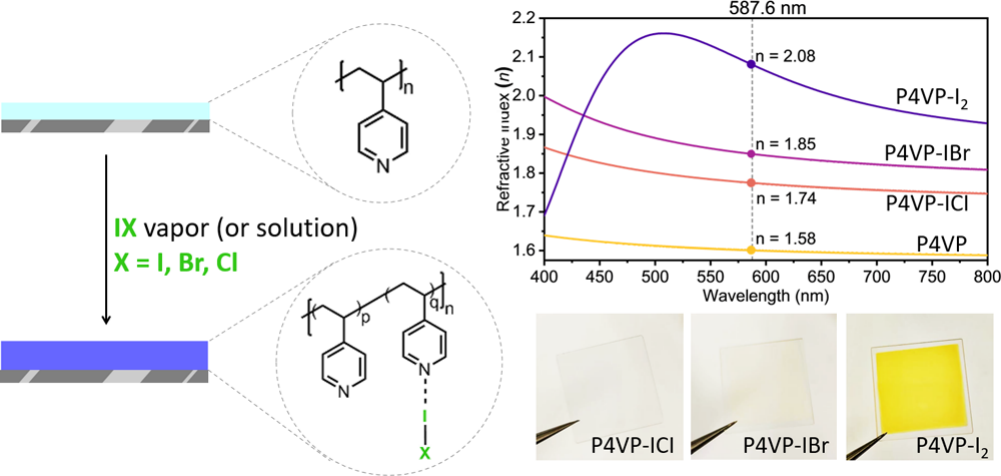

High refractive index polymers are essential in next-generation flexible optical and optoelectronic devices. This paper describes a simple synthetic method to prepare polymeric optical coatings possessing high refractive indexes. Poly(4-vinylpyridine) (P4VP) thin films prepared using initiated chemical vapor deposition are exposed to highly polarizable halogen molecules to form stable charge-transfer complexes: P4VP-IX (X = I, Br, and Cl). Fourier transform infrared spectroscopy was used to confirm the formation of charge-transfer complexes. Characterized by spectroscopic ellipsometry, the maximum refractive index of 2.08 at 587.6 nm is obtained for P4VP-I_2_. For P4VP-IBr and P4VP-ICl, the maximum refractive indexes are 1.849 and 1.774, respectively. By controlling the concentration of charge-transfer complexes, either through the halogen incorporation step or polymer composition through copolymerization with ethylene glycol dimethacrylate, the refractive indexes of the polymer thin films can be precisely controlled. The feasibility of P4VP-IX materials as optical coatings is also explored. The refractive index and thickness uniformity of a P4VP-I_2_ film over a 10 mm diameter circular area were characterized, showing standard deviations of 0.0769 and 1.91%, respectively.

## Introduction

Optical and optoelectronic devices must become increasingly adaptive, flexible, and compact to enable emerging applications, such as flexible electronics, wearable displays, augmented reality/virtual reality hardware, and flexible photovoltaics.^1,2^ As dielectric thin-film coatings are indispensable in optical design and utilized extensively in multilayered optical interference coatings, they too must be designed for flexibility. Besides interference applications, optical thin films are utilized as encapsulants for LEDs,^3^ microlens for complementary metal oxide semiconductor (CMOS) sensors,^4^ metalens,^5^ transparent electromagnetic shielding,^6^ and artificial cornea implants.^7^ Traditionally, these layers consist of inorganic compounds such as TiO_2_, Al_2_O_3_, and MgF_2_.^2^ However, several challenges arise when these brittle inorganic layers are integrated with soft, flexible, responsive polymeric substrates employed in next-generation optical devices. The difference in thermal and mechanical properties can lead to film fracture, delamination, and other mechanical failures.^8,9^ Moreover, the deposition of these inorganic layers through various physical vapor deposition techniques can lead to residual film stress or overheating that further compromises optical performance.^10,11^

Polymeric optical coatings are promising alternatives to traditional inorganic materials, as they possess similar mechanical properties to the flexible substrates and can accommodate a significant strain without fracture.^12^ Moreover, it has also been shown that the fracture developed under tension can close and essentially heal upon relaxation of stress. Despite these advantages, polymeric optical layers are limited by the range of readily accessible refractive indexes.^2^ In optical coating design, refractive index (*n*) is critical as it largely governs the number and thickness of individual layers in an interference coating.^13^ Typical refractive indexes of conventional optical polymers are in the range of 1.4–1.6; there are relatively few high refractive index polymer (HRIP) materials with a refractive index greater than 1.7.

As refractive indexes exceeding 1.7 are needed for an efficient optical design, the development of HRIPs is an active research field.^1,2^ HRIP development typically follows two approaches. The first is the synthesis of novel macromolecular structures incorporating elements and/or functional groups possessing high molar refractivities, such as sulfur,^14,15^ phosphorus,^16,17^ selenium,^18,19^ and aryl groups.^20^ In 2017, Jiang et al. reported a series of selenide-containing HRIPs, with *n* = 1.719 at 589 nm.^18^ Recently, Kim et al. reported a synthesis method for sulfur-containing polymer coatings with an RI of 1.97 by combining initiated chemical vapor deposition (iCVD) with reverse vulcanization.^21,22^ The second strategy involves the fabrication of polymeric nanocomposites.^1^ Typical nanoparticles selected as high RI dopants include TiO_2_,^23,24^ ZrO_2_,^25,26^ ZnS,^27,28^ and PbS.^29,30^ Metal or metal oxide materials are often employed as the inorganic fillers because synthetic methods to limit their characteristic lengths to below the Mie scattering limit (ideally <10 nm) are well-established.^31^ Ritchie et al. reported the preparation of polymer-titania nanocomposite coatings with an index of 1.936 at 635 nm via atomized spray plasma deposition.^32^

An important criterion for high index optical coating development is coating uniformity and smoothness. One of the key challenges for the above-mentioned strategies is inhomogeneity and phase separation with large concentrations of high index components. For example, incorporating large amounts of sulfur chains can lead to microphase separation, while agglomeration is a concern with high loadings of nanoparticles.^31^ Moreover, the development of optical coatings for flexible optics must consider the effect of processing steps on the substrates, which are often soft elastomeric materials with limited heat resistance. iCVD is a compelling processing technology given its ability to prepare films with exceptional control over thickness, composition, conformality, and spatial uniformity.^33−36^ Importantly, there is a limited heat transfer to the substrate, and substrates remain at low temperature throughout the iCVD process, enabling coatings to be prepared on temperature-sensitive substrates such as thermoplastic polyurethanes.^12,37−41^

In this work, we exploit iCVD to demonstrate a simple, efficient, scalable approach to prepare HRIP coatings with an RI as high as 2.08 at a wavelength of 587.6 nm. Through iCVD’s ability to control film compositions, we also demonstrate the modulation of RI within a wide range from 1.5 to 2.08 using a single copolymer chemistry. The approach utilizes the phenomenon of charge-transfer complex (CTC) formation between poly(4-vinylpyridine) (P4VP) and molecular I_2_ and interhalogen species. The pyridine residue functions as an electron donor (Lewis base), while iodine and interhalogens are electron acceptors (Lewis acids).^42,43^ It has long been recognized that electron transfer between pyridine and I_2_ results in a stable CTC with intriguing electrical and optical properties, which can be crystallized and isolated.^44^ This has likewise been observed between pyridine and the interhalogens, ICl and IBr.^45^ The favorable thermodynamics of the CTC formation promote the spontaneous incorporation of halogens into P4VP thin films at ambient temperatures, resulting in halogen-bearing polymer films with high refractive index.^46,47^ The benefits of using P4VP include its relatively high starting refractive index (prior to CTC formation, *n* = 1.58, measured at 587.6 nm) and well-established synthesis by iCVD.^34,35^ Although iCVD offers many advantages in the development of optical grade coatings, this HRIP fabrication approach can be readily adapted for P4VP prepared by other techniques like plasma-enhanced CVD, spin coating, and dip coating.^40^

## Results and Discussion

### Preparation of Polymeric Optical Coating

The Lorentz–Lorenz equation, which relates refractive index to microscopic material properties, guides the design of HRIPs.^1,2^ As framed in eq 1, it shows that the refractive index (*n*) is increased by maximizing the molar refraction (*R*_m_) and minimizing molar volume (*V*_m_).
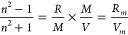
1

Among the elements and functional groups readily incorporated into synthetic organic polymers, the halogens, except fluorine, stand out for their high molar refractivities. For example, brominated methacrylate has been explored as high index waveguides.^48^ To incorporate halogens into polymeric optical layers, a two-step fabrication approach was employed, where a base polymer film was first synthesized by iCVD, and then halogens are incorporated by exposing the polymer films to either a halogen solution or saturated vapor. The CTC formation is thermodynamically favorable, and the maximum molar ratio of pyridine to IX in the CTC (X = I, Br, and CI) is believed to be 1:1,^45^ which will increase the P4VP’s refractive index to a large extent. In terms of substituents, I_2_ was the first substituent selected for incorporation into P4VP since it possesses the highest molar refractivity. IBr and ICl, which are stronger Lewis acids than I_2_, were also explored as alternative acceptors to establish a comprehensive understanding of chemical and optical properties of halogenated P4VP.

P4VP thin films were prepared in a custom-built iCVD chamber, depicted schematically in Figure S1. The iCVD process parameters were optimized to provide smooth, homogeneous, uniform films with typical deposition rates on the order of 10–20 nm/min, guided by previous iCVD studies of P4VP.^49^ The proposed chemical reaction mechanism constituting the iCVD process is provided in Figure S1b. The as-prepared P4VP thin films were vacuum dried overnight to fully ensure elimination of unreacted monomers. In the second processing step, three sets of P4VP thin films were treated by I_2_-hexane solution, IBr vapor, or ICl vapor, respectively. I_2_ was incorporated into P4VP thin films by immersing the film into a 0.05 mol/L I_2_-hexane solution; the exposure duration varied from 0.5 to 6 h. The formation of the CTC is spontaneous. The films were then rinsed with fresh hexanes to remove free, non-complexed I_2_. Hexane is an excellent solvent as it readily dissolves the nonpolar I_2_ without otherwise affecting the P4VP film. IBr and ICl are incorporated into the film through a vapor exposure process as their increased polarity makes them insoluble in hexane. A clean, dry P4VP film supported on the silicon wafer was placed inside a sealed glass container with a small vial containing 300 μg of IBr/ICl. The sealed container was placed in glass under 40 °C (for IBr) or 30 °C (for ICl) for a period from 0.5 to 6 h. After the halogen compound treatment, all samples were flushed with an argon flow for 1 min to remove residual non-complexed halogens. During both processes, the reflected color of the films coated on the silicon wafer evolves with exposure duration, suggesting changes in film thickness and optical properties due to halogen incorporation. Importantly, the films appear uniform and reflective with no obvious scattering or haze, suggesting that the film is continuous and homogeneous.

**Figure 1:**
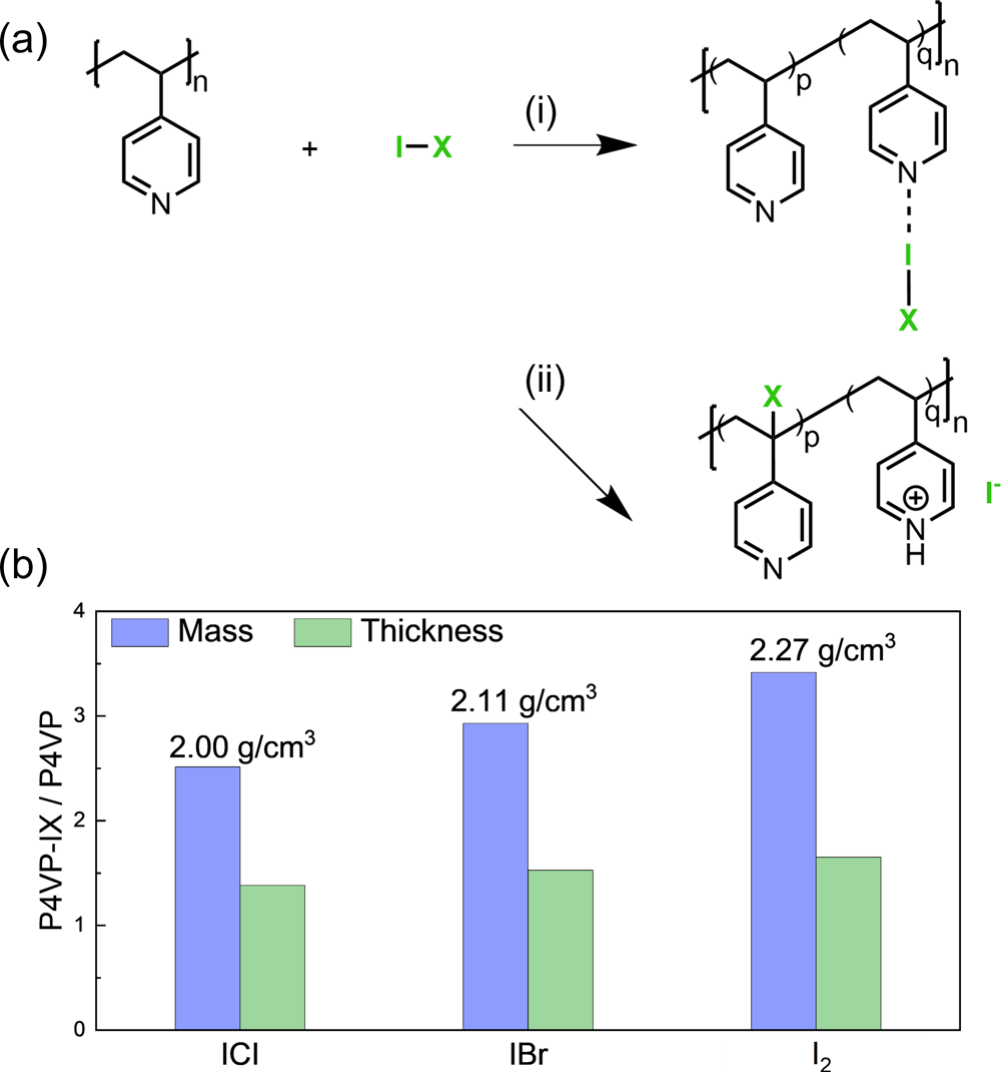
(a) Reaction scheme depicting the (i) formation of polymeric CTC and (ii) potential side reaction involving halogenation of the aliphatic backbone. (b) Measured changes in physical properties of P4VP-IX thin films (mass and thickness) with an estimated film density after maximum halogen incorporation. The *y*-axis represents the ratio of the measured properties in the complexed P4VP relative to the base P4VP.

The effect of halogen incorporation on the film density was studied by measuring the film thickness and mass before and after halogen incorporation. As reported in Figure 1b, the mass of P4VP films increased by 241.6 ± 14.2, 193.0 ± 10.4, and 149.5 ± 7.7% with the maximum exposure to I_2_, IBr, and ICl, respectively. This mass increase is consistent with the formation of a 1:1 adduct between P4VP and molecular IX (X = I, Br, or Cl). Assuming the mass increases stem solely from the incorporated halogen species, the maximum molar ratio of IX to pyridine is 1.0:1.0, 0.99:1, and 0.98:1 in P4VP-I_2_, P4VP-IBr, and P4VP-ICl, respectively. Meanwhile, the thickness of P4VP-I_2_ increases by 65.2% of the original P4VP’s thickness, with less film expansion for IBr and ICl (see Figure 1b). According to the measured thicknesses and masses, the densities of P4VP-I_2_, P4VP-IBr, and P4VP-ICl were determined to be 2.3, 2.1, and 2.0 g/cm^3^, respectively. These measured densities are substantially increased relative to P4VP homopolymers with a density of 1.10 g/cm^3^.^50^ The large mass density is a function of the compact structure of the P4VP-halogen complex and contributes to the high refractive index of the films through the molar volume term in the Lorentz–Lorenz equation (eq 1).

The mass measurements only provide information about the maximum CTC concentrations in the films. FTIR was used to show that the CTC concentration in P4VP-I_2_ can be controlled through the length of exposure to the I_2_ solution. A series of FTIR spectra of P4VP-I_2_ thin films as a function of treatment time are shown in Figure S5. The absorbance at 1609 cm^–1^ in all P4VP-I_2_ spectra is assigned to the quadrant stretch of the pyridine ring in the complex. It is shifted from 1596 cm^–1^ in the uncomplexed P4VP due to electron redistribution in the pyridine ring toward the halogen cation in the CTC. The absorbances for complexed and uncomplexed P4VP were deconvoluted, and the ratios of the integrated absorbances are reported in Table S1. The ratio of the complexed-to-uncomplexed absorbances increases from 0.69 to 1.40 when the iodine solution treatment was extended from 1 to 12 h—a 203% increase. Unfortunately, quantitative estimates of CTC concentrations are not possible since the molar absorptivity of the complexed and uncomplexed quadrant modes may differ, but their ratios as a function of time in Table S1 validate that the relative CTC concentrations can be manipulated through the halogen exposure.

### Chemical Composition Analysis of P4VP-IX

**Figure 2:**
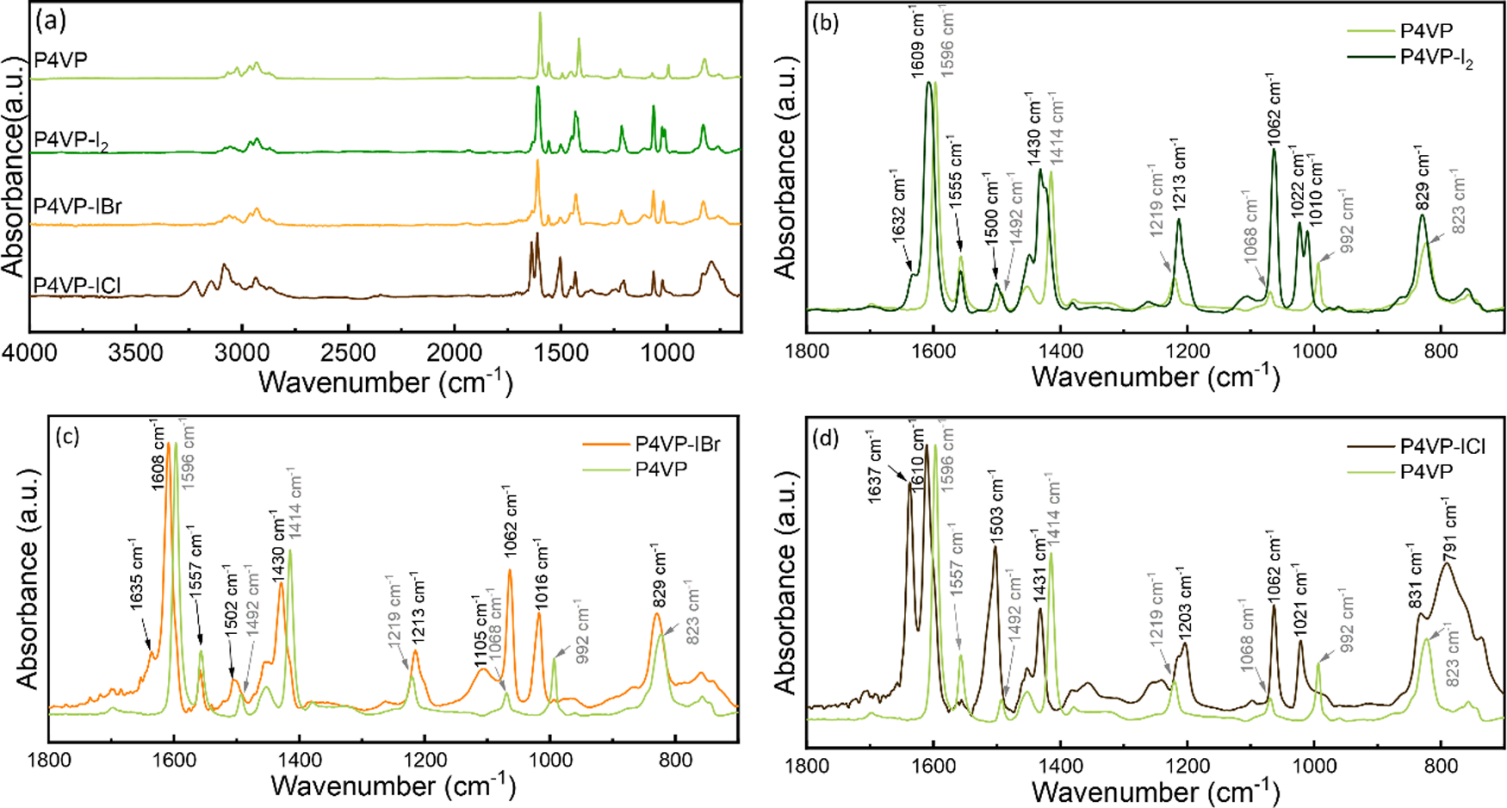
Fourier transform infrared spectroscopy (FTIR) spectra of (a) P4VP and the CTC thin films with magnification of the fingerprint regions for (b) P4VP-I_2_, (c) P4VP-IBr, and (d) P4VP-ICl.

The FTIR spectra of P4VP and P4VP-IX are reported in Figure 2. The spectra for each complex over the entire mid-infrared region are provided in Figure 2a, and the fingerprint regions of P4VP and P4VP-I_2_, P4VP-IBr, and P4VP-ICl are compared in Figure 2b–d. While key vibrational modes of I–Br, I–Cl, and polarized I–I bonds are located in the far-IR region (<400 cm^–1^) and were not measured using a typical mid-infrared spectrometer, characteristic vibrational modes of the pyridine ring influenced through complexation are found from 1700 to 700 cm^–1^.^51^ For as-deposited P4VP, at least seven characteristic vibrational modes of the pyridine ring are observed. The two bands at 1596 and 1555 cm^–1^ are assigned to the quadrant stretch mode, and the bands at 1492 and 1414 cm^–1^ are assigned to the semicircle stretch mode. These four bands are clear indication of the intact pyridine ring. The absorption bands at 1068 and 1219 cm^–1^ are assigned to the in-plane and out-of-plane ring C–H bending, respectively, and the band at 992 cm^–1^ is assigned to the radial breathing mode of pyridine.^52^

Similar bands were also observed in the P4VP-IX spectra, indicating retention of the P4VP chemical structure. These characteristic modes exhibit shifted energies and variable intensities compared with the modes of the pure P4VP and are consistent with previous reports of these complexes.^51,53^ FTIR analysis largely supports the formation of CTC in these films, as depicted in reaction (i) in Figure 1a. In P4VP-I_2_, the quadrant and semicircle stretch modes shift to higher energies from 1596 to 1609 and from 1414 to 1430 cm^–1^, respectively. This is caused by the electron redistribution of the pyridine ring toward the halogen cation in the CTCs. This observation also reveals that the pyridine ring’s C–N/C–C bonds are strengthened upon the formation of the charge-transfer compounds.^54^ Notably, the absorption intensities at 1068, 1219, and especially 992 cm^–1^ increased significantly with the addition of iodine content.

The changes in P4VP-IBr and P4VP-ICl shown in Figure 2c,d are largely consistent with complexation. However, in each of these spectra, there is a shoulder on the quadrant stretch peak (1609 ± 1 cm^–1^), which is centered at 1635 ± 3 cm^–1^. This peak is characteristic of quaternized pyridinium, the formation of which occurs through an α-H abstraction described in reaction (ii) in Figure 1a. In P4VP-I_2_ and P4VP-IBr, the absorbance of the pyridinium is quite small, indicating a limited extent of side reaction. However, in P4VP-ICl, the intensity of the pyridinium mode is comparable to the quadrant mode. Additionally, evidence of the formation of protonated pyridinium is also observed in Figure 2d in the 2700–3400 cm^–1^ region. Coupled with the new vibrational modes at 791 cm^–1^ assigned to the C–Cl stretch on the aliphatic backbone, it can be concluded that an appreciable side reaction has occurred due to the greater reactivity of the Cl radical. The fraction of the side reaction relative to complex formation has not been quantified. This side reaction is described in greater detail in a previous study by Li et al.^40^

### Optical Properties of P4VP-IX

**Figure 3:**
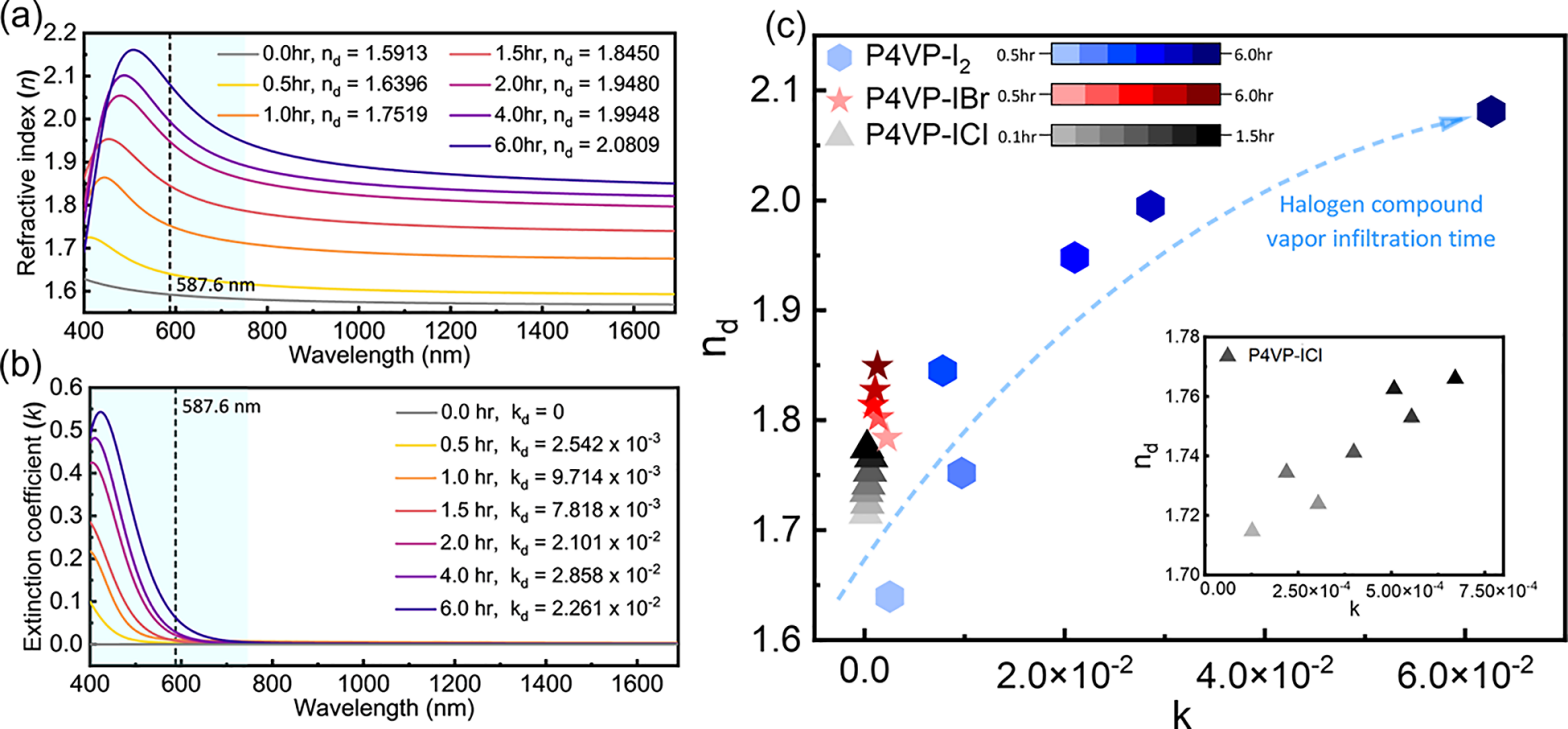
(a) Refractive index and (b) extinction coefficient spectra of P4VP-I_2_ as a function of iodine-hexane solution treatment time. (c) Extinction coefficients and refractive indexes measured at 587.6 nm for a series of P4VP-I_2_, P4VP-IBr, and P4VP-ICl thin films with varying extents of halogen incorporation. The duration of halogen exposure (either in solution or vapor) is indicated by the color scale.

The optical properties of P4VP-IX films were extensively characterized by spectroscopic ellipsometry. The refractive index (*n*) and extinction coefficient (*k*) spectra of P4VP-I_2_, P4VP-IBr, and P4VP-ICl as a function of treatment times are plotted in Figure 3 and Figure S2. These plots were derived from ellipsometry models. Models based on the Cauchy equation and Lorentz oscillator equations were used to fit the experimental ellipsometric data from P4VP-IX films. The Cauchy model generally describes refractive indexes of transparent materials with negligible absorption, while the Lorentz oscillator model accounts for absorption through a series of wavelength-dependent oscillators. The Cauchy model adequately describes P4VP at wavelengths above 400 nm, but the Lorentz oscillator model is needed to comprehensively describe the P4VP-IX films, suggesting that there is non-negligible absorption in these films. Moreover, additional model refinement processes to optimize model performance were examined, including an index gradient normal to the interface and non-uniformity in the plane of the films. These additional model refinements did not significantly improve the mean square error of the fitting of the optical parameters, revealing that the film is spatially homogeneous and without a gradient in the index of refraction normal to the substrate. Complex formation appears to be uniform throughout the film. A detailed study regarding the homogeneity and uniformity of the film is discussed later.

Figure 3a provides the refractive index spectra. For the baseline P4VP prior to iodine incorporation, the index of refraction measured at 587 nm is 1.591. Normal dispersion of the refractive index is observed, where the index decreases with wavelength, and the extinction coefficient above 400 nm was zero (Figure 3b). The refractive index of the treated polymer films is directly correlated to the exposure duration to the iodine solution, revealing the relationship between CTC concentration and index. The same trend is observed in the films exposed to ICl and IBr vapor (Figure S2). With iodine incorporation, index of refraction dispersion becomes anomalous (i.e., there is a maximum in the index) and the extinction coefficient becomes significant, as revealed in Figure 3b. Refractive index and extinction coefficients are dependent parameters, linked through the Kramers–Kronig relationship,^13^ and the anomalous dispersion is a result of the significant absorption in the complexed films. The index of refraction and extinction coefficient (*k*) spectra of P4VP-IBr and P4VP-ICl are reported in Figure S2. The optical properties of these complexes show a similar correlation to the concentration of incorporated halogens, but normal index dispersion is observed in the visible region as the absorption maxima are in the ultraviolet.

Among the three materials, the P4VP-I_2_ film with the largest index of refraction of 2.08 also possesses the highest *k*, reaching a value of 6.26 × 10^–2^ at 587.6 nm after 6 h. The P4VP-IBr film with an *n* of 1.85 exhibits a much smaller extinction coefficient of 1.30 × 10^–3^—over an order of magnitude lower than P4VP-I_2_. P4VP-ICl possesses an almost negligible maximum extinction coefficient of 2.61 × 10^–4^. The maximum value of *k* in P4VP-I_2_ occurs at around 423 nm. Figure 3b also shows that the absorption maxima of P4VP-I_2_ are centered at lower wavelengths when less I_2_ is incorporated into the film (i.e., shorter treatment times). This is consistent with a seminal study by Reid and Mulliken, which showed that the visible absorption maximum of I_2_ in the pyridine-I_2_ complex shifts to lower wavelengths upon adding pyridine in large excess.^44^ The absorption maxima at 423 nm in P4VP-I_2_ are attributed to the electronic transitions in I_2_, which is shifted from 520 nm for I_2_ vapor or iodine solutions in non-complexing, inert solvents. This significant shift is a result of the dative nature of the complex and the resulting dipole that develops within the I_2_ molecule.^43,44,55^ Electronic transitions of the CTC are expected to absorb at much lower UV wavelengths (<350 nm) but are masked by the absorption of pyridine and were not reconciled spectroscopically in this study.^44,56^

**Figure 4:**
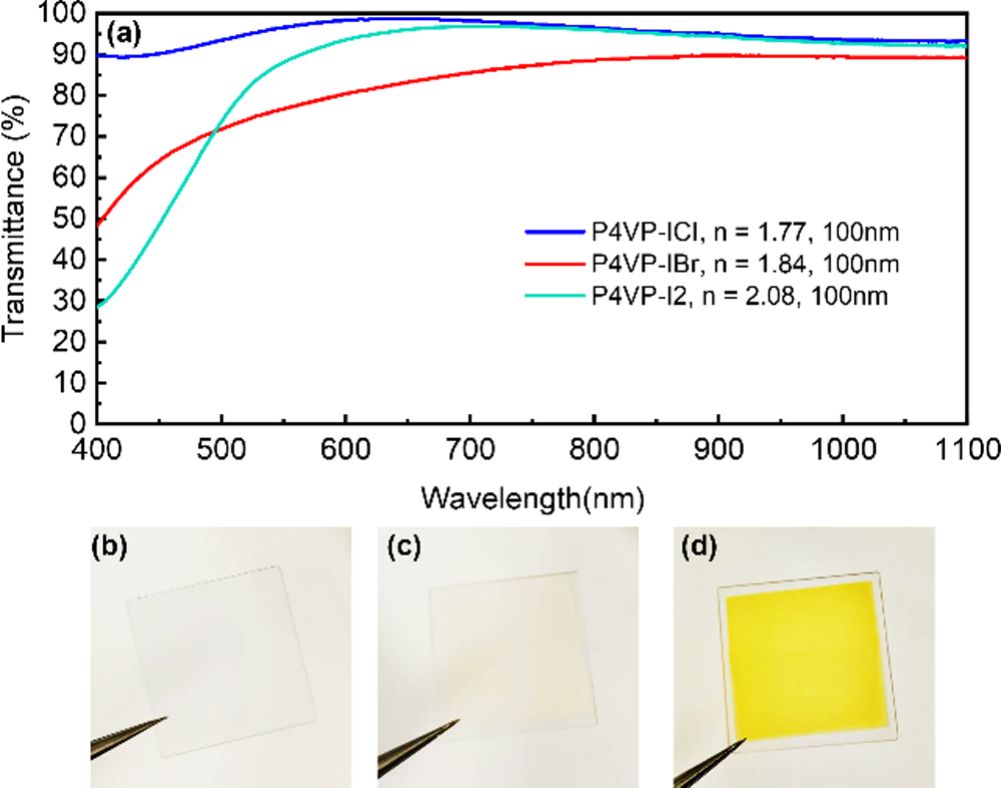
(a) Transmission spectra of 100 nm thick P4VP-ICl/IBr/I_2_ thin films. Digital photographs of 1000 nm thick (b) P4VP-ICl, (c) P4VP-IBr, and (d) P4VP-I_2_ thin films coated on quartz substrates.

To further understand the effect of the optical absorption within the complexes, transmittance of the films was also measured. The transmittance of 100 nm P4VP-I_2_, P4VP-IBr, and P4VP-ICl coated on quartz in a wavelength range of 400–1100 nm is plotted in Figure 4a. The transmittance of P4VP-IX coincides with the analysis of complex index of refractions of P4VP-IX. Transmittance was highest in P4VP-ICl, while significant absorbance at lower visible wavelengths is observed in P4VP-I_2_. Digital photographs of the P4VP-IX films (1000 nm thick) are shown in Figure 4b–d. P4VP-I_2_ appears brownish yellow, while P4VP-IBr appears slightly yellow, and P4VP-ICl is almost transparent. The observations in their color differences are consistent with the transmittance measurements.

Despite the significant visible absorption, P4VP-I_2_ have great potential in applications like multilayered waveguides, CMOS sensors, and IR lenses. The transmittance of P4VP-I_2_ thin films is considered acceptable for these applications, as shown by its average transmittance of 82% in the visible region (400–800 nm) for a 100 nm thick film. To reduce visible absorption, the strategy employed in this study was to modify the Lewis acid (acceptor) component in the complex. The strength of Lewis acidity in the halogen molecules is ICl > IBr > I_2_.^43,57^ The stability of the CTC increases with the Lewis acidity of the acceptor, further shifting the electronic transition within the acceptor molecules. The absorption maxima of ICl shift from 459 nm as a vapor or in inert solvents to less than 300 nm in P4VP-ICl.^40,57^ Thus, the coating appears transparent due to the limited absorption at visible wavelengths, but the compromise, of course, is the lower index of refraction. To maximize index of refraction while minimizing visible absorption, one strategy is to engineer the donor component to strengthen the CTC with I_2_ and blue-shift the iodine absorption further. This can be achieved by increasing the basicity of the nitrogen heteroatom in pyridine through electron-donating substituents on the ring.^57,58^ Alternatively, polymer compositions with stronger basic pendant functional groups can be explored, recognizing that the molar volume is also critical to a film’s index of refraction.^56^

Besides refractive index and optical transparency, optical dispersion is another important parameter in designing HRIPs for various optical applications. The chromatic aberration of the P4VP-IX thin films was compared using the Abbe number, which describes the dispersion in the refractive index spectrum. By definition, the Abbe number is calculated using *n* measured at 486.1, 587.56, and 656.3 nm (corresponding to Fraunhofer F, d, and C spectral lines), respectively.^59^ A low Abbe value indicates more chromatic aberration of the lens. The Abbe number of P4VP-IX with respect to refractive indexes is plotted in Figure S3. For P4VP-ICl, the Abbe number varies from 17.2 to 19.8 as the RI varies from 1.71 to 1.77. For P4VP-IBr, the Abbe number varies from 12.2 to 14.5 as the RI varies from 1.78 to 1.85. Finally, in P4VP-I_2_, the Abbe number varies from 6.0 to 31.0 as the RI varies from 1.59 to 2.08. Ideally, the Abbe number should be above 40, which is the threshold for human eyes to detect the chromatic aberration.^59^ However, there is a trade-off between the refractive index and Abbe’s number as the refractive index increases. Future effort should develop strategies to reduce dispersion in these materials.

### Index Control through Copolymerization

**Figure 5:**
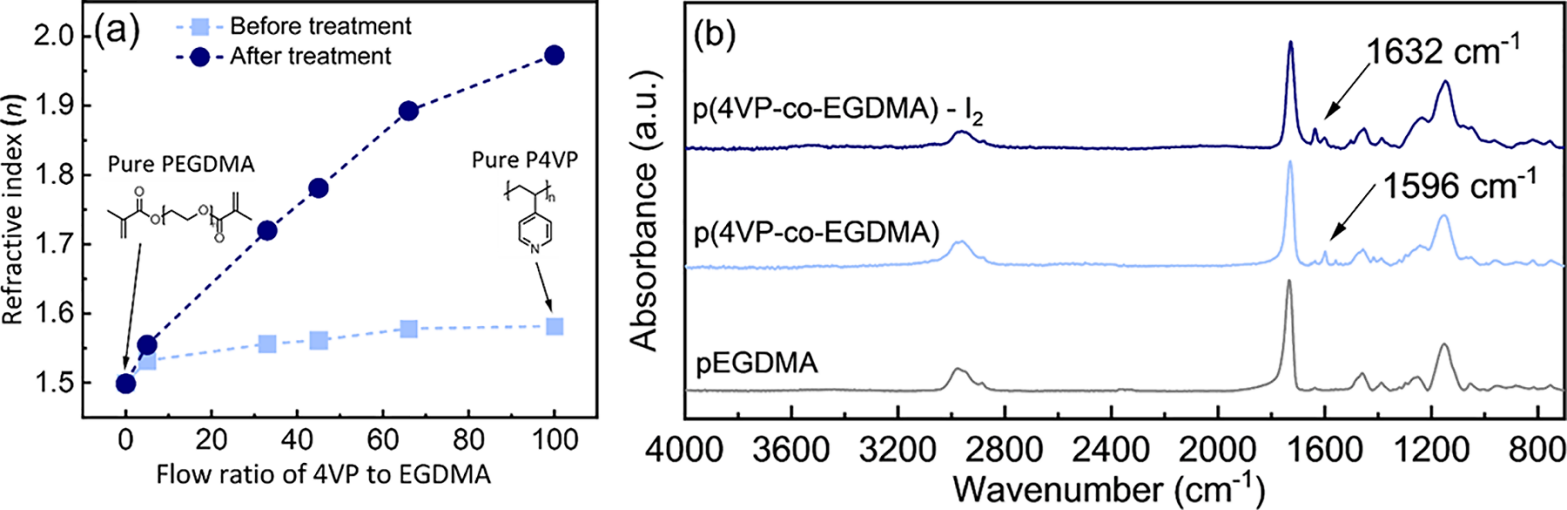
(a) Refractive indexes of p(EGDMA-*co*-4VP) thin films as a function of the flow ratio of 4VP and EGDMA vapor. (b) FTIR spectra of untreated and iodine vapor treated p(4VP-*co*-EGDMA) thin films.

**Figure 6:**
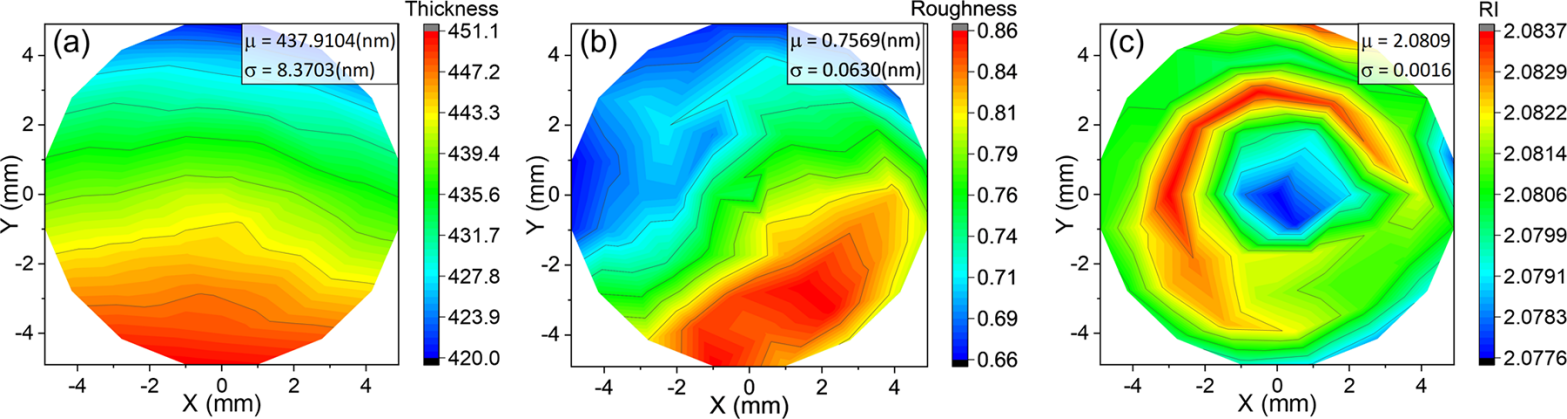
Maps of (a) thickness, (b) roughness, and (c) refractive index of P4VP-I_2_ over a 10 mm diameter circular area. μ and σ represent the mean and standard deviation, respectively, of the physical property over the mapped area.

As demonstrated above, the optical properties of the P4VP-IX thin films can be adjusted by controlling the concentration of CTCs through the halogen exposure process. Another approach is to copolymerize 4-vinylpyridine (4VP) with a comonomer that is inert to the halogen complexation. The benefit of this approach is that it allows further modulation of the refractive index, expanding the tunable range through copolymer composition. Ethylene glycol dimethacrylate (EGDMA) is one example of an inert comonomer, as preliminary trials showed that homopolymer films of poly(ethylene glycol dimethacrylate) do not form CTCs with iodine or its interhalogens. Therefore, a series of P(4VP-*co*-EGDMA) copolymers were prepared with varying compositions and treated by I_2_-hexane solution. I_2_ is selected because P4VP-I_2_ yields the higher index of refraction relative to IBr and ICl; thus, it is expected to demonstrate a wider range of RI tunability. Figure 5a shows that as the fraction of 4VP in the precursor gas feed increases, the refractive indexes of the base copolymer films increase from around 1.50 to 1.58. This is correlated to the concentration of 4VP in the film. The relationship between the copolymer composition and the refractive index can be described through volume-averaged effective medium approximation.^60^ Additionally, P(4VP-*co*-EGDMA) is a microscopically homogeneous statistical copolymer, and microphase separation is not observed. The copolymer films retain their uniform, transparent appearance, as demonstrated by uniformity characterizations presented in Figure 6 and Figure S4. Upon exposure to I_2_-hexane solution for 4 h, the relative increase and ultimate refractive index is a function of the 4VP composition, monotonically increasing with the concentration of 4VP (Figure 5a). This approach enables the refractive index to be tuned over a wide range from 1.5 to 1.98, providing a single material solution for a wide range of optical design requirements.

The FTIR spectra of PEGDMA homopolymers, P(4VP-*co*-EGDMA) copolymers, and P(4VP-*co*-EGDMA)-I_2_ thin films are presented in Figure 5b. The highest peak centered at 1722 cm^–1^ present in all three spectra is characteristic of the carbonyl stretch of EGDMA. The characteristic peaks of the quadrant stretch of 4VP centered at 1596 cm^–1^ are observed in the FTIR spectra of the P(4VP-*co*-EGDMA) copolymer, confirming the copolymerization. The large difference in absorbances for these two characteristic peaks is a consequence of their respective absorptivity (e.g., the carbonyl stretch mode absorbs strongly, while the quadrant mode is relatively weak). In the spectrum of the P(4VP-*co*-EGDMA)-I_2_ thin film, both characteristic peaks of 4VP and EGDMA are observed. The carbonyl stretch absorbance remains unchanged as the EGDMA component is inert to halogen complexation. Additionally, characteristic shifts in the 4VP vibrational modes (e.g., quadrant stretch blue-shifts to 1609 and 1596 cm^–1^) are observed, indicating that the CTCs can form on the 4VP moieties in the copolymer and that the difunctional EGDMA crosslinker (and comonomer) does not preclude CTC formation. Also, a new peak at 1632 cm^–1^ representing the pyridinium was observed in the spectra of the treated copolymer film, indicating that the side reaction observed between I_2_ and P4VP also occurred between I_2_ and copolymer. The effect of EGDMA on the diffusion kinetics of the halogens into the film has not been quantified.

### Surface Morphology Characterization

Refractive index and thickness uniformity are of great importance in optical coating fabrication, as the consistency of optical performance across the optics’ entire surface is dependent upon the homogeneity of each layer. Thickness variation can lead to a deviation in optical interference. Second, defects and high roughness brought by composition inhomogeneity can cause scattering or haze. To ensure that optical layer stacks could achieve the targeted performance, coating materials must possess high compositional and thickness uniformity and low surface roughness. Accordingly, to examine the practicality of P4VP-IX as optical coatings, the uniformity of film thickness, roughness, and refractive index of coatings over a 78.5 mm^2^ circular area (10 mm diameter) was characterized by mapping ellipsometry.

The thickness, roughness, and refractive index maps of P4VP-I_2_ are presented in Figure 6, and the corresponding maps for P4VP-IBr and P4VP-ICl can be found in Figure S4. A nominal film thickness of 400 nm was selected for this analysis; thinner films prepared by iCVD are expected to have superior uniformity. The P4VP-I_2_ is uniform with an average thickness of 437.9 ± 8.4 nm, with a standard deviation of only 1.91% for the entire area. P4VP-IBr and P4VP-ICl are also comparably uniform with the measured thickness being 409.8 ± 6.1 and 403.1 ± 4.7 nm, respectively. Surface roughness was estimated by adding an additional “effective” roughness layer in the existing model. All three P4VP-IX exhibit extremely low surface roughness, with the maximum surface roughness being less than 1 nm over a 1 cm diameter.

Notably, the mean refractive index of P4VP-I_2_, which is a function of the film composition, is 2.0809 ± 0.0016, with the standard deviation being 0.0769% of the mean. P4VP-IBr and P4VP-ICl also show excellent RI uniformity, with average RI being 1.8490 ± 0.0023 and 1.7743 ± 0.0012, respectively. As demonstrated previously, complex formation appears to be uniform, as adding a gradient variable along the depth of the film does not improve the accuracy of the model. Characterizing the RI and thickness variation over the entire sample area further confirms that the film is also uniform along the *x*–*y* plane, indicating the homogeneous nature of the material, which can be attributed to the efficient CTC formation through solvent treatment/vapor infiltration processes.

Comparing the thickness, roughness, and RI maps of the complexed film to the base P4VP film (Figure S7) reveals that the halogenation process does not significantly compromise the thin-film morphology. Before and after halogenation, the surface roughness remains below 1 nm, indicating a highly smooth surface. Visual observations of all samples were consistent with smooth, uniform, high-quality films, as are expected of iCVD.

### Thermal Stability

**Figure 7:**
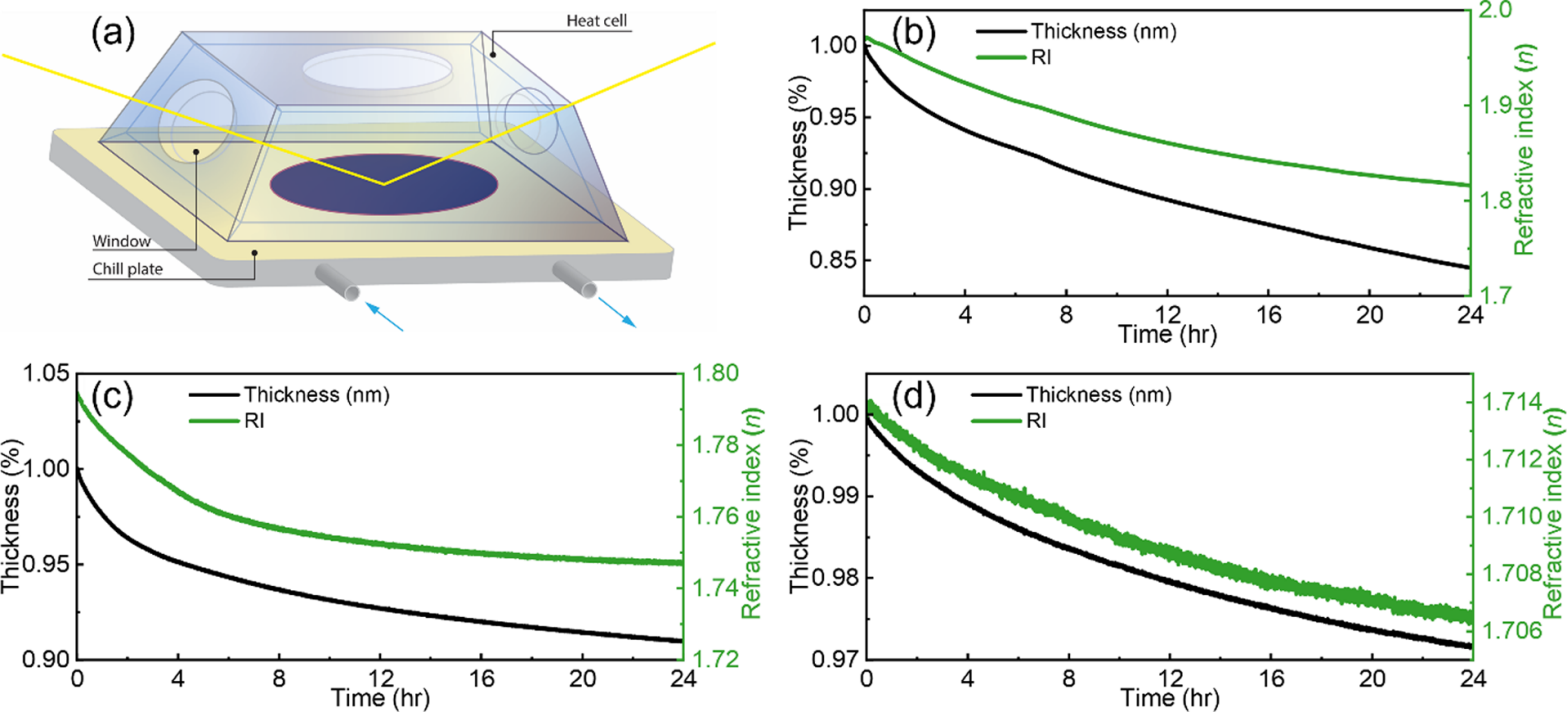
(a) Schematic of the ellipsometer thermal cell used to characterize the thermal stability of P4VP-IX coatings. Argon gas purged the sample chamber at a flow rate of 20 sccm (not shown in the schematic). Thickness and refractive index of (b) P4VP-I_2_, (c) P4VP-IBr, and (d) P4VP-ICl thin films measured in situ over 24 h at 20 °C.

Another important consideration is the environmental stability of the P4VP-IX coatings. Thermal stability is critical as coatings may undergo high-temperature excursions during further device processing. They must also remain stable over a wide range of temperatures. Though the Gibbs energy of formation of these complexes is favored, the interactions are expected to be much weaker than covalent or ionic bonds.^40^ The environmental stability tests were performed by monitoring the optical parameters and film thickness of the sample in situ at 20 °C for over 24 h. Ellipsometric data were collected every 30 s as the cell was continuously purged with dry argon. A schematic of the experimental setup is provided in Figure 7a.

The thickness and refractive index changes of P4VP-IX during the 24 h thermal stability tests are plotted in Figure 7b–d. P4VP-I_2_ experienced a reduction of 15.5% in thickness and 7.90% in RI over 24 h. This volume and RI loss can be explained by CTC dissociation, followed by evolution of the I-X from the film. The N–I bond in P4VP-I_2_ CTC is much weaker compared to regular covalent bonds, such that the P4VP-I_2_ CTCs can be easily decomposed and release I_2_ with heating/argon purge. The FTIR spectra of P4VP-I_2_ films after discrete time intervals of nitrogen purging, up to 24 h, are shown in Figure S6. The intensity of the 1609 cm^–1^ absorbance, a measure of P4VP-I_2_ CTC, relative to the non-complexed absorbance decreases with longer purge times. The ratio of the complexed-to-uncomplexed absorbances is from 1.96 to 0.78 over 24 h of nitrogen purging, supporting the conclusion that the reduction in index and thickness is due to CTC dissociation. However, CTC dissociation is not incomplete, and appreciable complex remains after 24 h. Similar mass and thickness reduction of P4VP-I_2_ films has also been previously observed when the films are placed in vacuum.^40^

Interestingly, P4VP-IBr and P4VP-ICl exhibit much improved thermal stability. While P4VP-IBr shows a thickness reduction of 9.0% and an RI reduction of 2.6%, P4VP-ICl exhibits a reduction of only 2.8% in thickness and 0.43% in RI over 24 h. This can be explained by the increased Lewis acidity of IBr and ICl compared with I_2_, resulting in more stable CTCs with the pyridine ring. For P4VP-ICl, the more stable physical properties can be partially attributed to the side reactions (Figure 1b) that result in stronger covalent and ionic bonds, which are expected to be more irreversible. The limited thermal stability performance of P4VP-IX will likely restrict their potential applications. Encapsulation of the layers with barriers is one potential solution. It is expected that the highly crosslinked polymeric films, such as 1*H*,1*H*,6*H*,6*H*-perfluoro-1,6-hexyl diacrylate, can function as flexible barrier layers, mitigating halogen loss and extending the temperature resistance of these coatings. Approaches that incorporate halogens through strong covalent or ionic bonds are likely to provide superior thermal stabilities.

## Experimental Section

### Deposition of P4VP and P(4VP-*co*-EGDMA) Thin Films

All polymer thin films were prepared by iCVD using a custom-built vacuum deposition system.^12^ The vacuum chamber’s pressure was maintained at 500–1500 mTorr using a pressure transducer (Brooks Instrument, CMX100) and a feedback-controlled downstream throttle valve (MKS Type 53B). The substrate temperature was controlled by an external recirculating chiller/heater unit (VWR, AD15R-40-V11B) and a liquid-filled aluminum cold plate (Wieland MicroCool). The power was supplied to the nichrome filament by a DC power supply (TDK-Lambda, GEN150-5). The thickness of the deposited film is monitored on a real-time basis using the laser interferometer (Thorlabs, PM100USB). Mass flow controllers (MKS Instrument, Type 1179) were utilized to regulate the flow of carrier gas, diluent gas, and initiator.

The precursors 4VP and EGDMA are introduced into the chamber by sparging liquid reservoirs with Ar carrier gas. The glass jars containing monomers were submerged in a water bath and maintained at 25 °C to provide stable vapor pressure and flow rates. The initiator di-*tert*-butyl peroxide (TBPO) is fed into the chamber without carrier gas due to its relatively high vapor pressure at room temperature. High-purity Ar (5 sccm) was employed to deliver 4VP (Fisher Scientific, 95% purity) vapor into the chamber. Di-*tert*-butyl peroxide (2 sccm) (TBPO, Fisher Scientific, 99% purity) was utilized as the initiator. For P(4VP-*co*-EGDMA) copolymer deposition, EGDMA carried by Ar flow was fed into the chamber, with the rate of carrier flow varying from 5 to 30 sccm. To improve thickness uniformity, an additional 10 sccm of Ar flow was added to dilute the reaction mixture inside the chamber. After deposition, the samples were placed in a vacuum oven (VMR, 12.5 L unit) overnight to remove any residual absorbed in the film and prevent water absorption.

### Halogenation of P4VP Thin Films via Solvent Treatment/Vapor Infiltration Processes

Three sets of P4VP thin films were treated by I_2_-hexane solution, IBr vapor, or ICl vapor, respectively. An incubator (VWR, Low Temperature Personal Incubator) was used to provide a stable temperature environment for the halogenation treatment. For P4VP-I_2_, I_2_ was added to the P4VP film via a solution treatment. The film is submerged in a 0.08 mol/L I_2_-hexane solution within a sealable glass container, which was then placed in the incubator set at 25 °C. After I_2_-hexane solution treatment, the treated film is flushed with pure hexanes for 1 min. P(4VP-*co*-EGDMA)-I_2_ thin films are also prepared by treating as-prepared copolymer thin films with I_2_-hexane solution. A series of P(4VP-*co*-EGDMA) thin films with different composition ratios were submerged in 0.08 mol/L I_2_-hexane solution for 4 h.

Due to IBr and ICl’s limited solubility in hexanes, P4VP-IBr and P4VP-ICl were prepared using vaporized IBr and ICl. For ICl vapor treatment, red light is used as the only lighting source since ICl is highly photosensitive.^40^ A piece of the annealed P4VP-coated silicon wafer was placed inside a sealed glass container, along with a 1 mL glass vial containing a drop of IBr liquid (300 μL) or ICl liquid (300 μL). The glass jar is then sealed and placed inside an incubator with temperature kept at 40 °C (for IBr treatment) or 30 °C (for ICl treatment). For ICl, the vapor treatment time varies from 5 min to 2 h, depending on the targeted degree of halogenation. The treatment time was limited to 2 h for ICl to prevent the film from degradation caused by hydrogen halides formed due to the strong side reaction between P4VP and ICl.^40^

### Density Characterization

To calculate the density of the P4VP-IX with a maximum degree of halogenation, the thickness and weight of samples before and after halogenation treatment were measured by ellipsometry and microbalance (Mettler Toledo XPR6UD5, 0.5 μg accuracy), respectively. The accuracy of the balance is sufficient for the thin-film samples with masses on the order of 1 mg.

The weight of the polymer thin-film coating prior to halogenation is calculated by subtracting the weight of the uncoated substrate (clean silicon wafer) from the coated substrate. After halogenation, residual uncomplexed halogens were extracted by rinsing with pure hexanes or blowing a stream of Ar over the surface. The samples were also placed in a desiccator for 24 h to remove any physically absorbed halogen species. The samples were then reweighed. The volume of the films was calculated using the substrate area and measured film thickness (see below for details on ellipsometry measurements).

### Composition Characterization

The FTIR and UV–vis spectra were collected to confirm the formation of P4VP-IX and quantify the optical transmittance. The FTIR spectra were acquired on polymer thin films coated on silicon wafers (Thermo Fisher Scientific, Nicolet iS-50). For all measurements, the resolution was set to 4 cm^–1^ and a total of 64 scans were integrated. The spectra of the silicon substrates were subtracted from the spectra for the film; the resulting absorbance spectra were baseline corrected. To provide a more straightforward comparison between P4VP and P4VP-IX, all spectra in Figures 4 and 6 are normalized.

### Optical Property Characterization

The optical constants (refractive index and extinction coefficient), film thickness, and roughness were characterized by spectroscopic ellipsometry (J.A. Woollam, RC2). The refractive index and thickness of P4VP, P4VP-IX, P(4VP-*co*-EGDMA), and P(4VP-*co*-EGDMA)-I_2_ single-layer films were derived from fitting the raw ellipsometry data with customized optical models. The ellipsometry data were collected at the wavelength range from 400 to 1690 nm with an incident angle of 65°. The raw ellipsometric data was fit to optical models using the proprietary CompleteEASE software licensed from the J.A. Woollam Company. The Cauchy model was selected for pure PEGDMA and P(4VP-*co*-EGDMA) due to their negligible absorption over the measured wavelength range. The built-in “General Oscillator” model, which accounts for absorption through a series of wavelength-dependent oscillators, was used for fitting raw data of P4VP-IX and P(4VP-*co*-EGDMA)-I_2_. The roughness of the sample is calculated based on the refractive index change at the interface between the sample surface and air. To validate the uniformity of the sample, thickness, roughness, and refractive index information are collected in over 20 different locations within each sample. The selected data collection sites are evenly distributed along the sample surface. The collected data of each parameter at an incident angle of 65° are plotted in the form of a map. The average value and the standard deviation were calculated. Transmission measurements were conducted on 100 nm P4VP-IX films coated on quartz wafer using a UV–vis spectrophotometer (Thermo Scientific, Evolution 300). The spectrum was collected from 400 to 1100 nm, with a 1 nm interval. For all measurements, the spectra of the quartz substrates were first collected and subtracted to eliminate losses associated with the substrate.

## Conclusions

In this work, a straightforward method for creating HRIP thin-film coatings is described. Polymer thin films are prepared via iCVD and then infiltrated with halogen compounds through simple solution or vapor treatments. Halogen elements are immobilized in the polymer matrix via the formation of a CTC between the Lewis acidic molecular halogen and the Lewis basic pyridine moieties in P4VP. With the maximum degree of iodination, the refractive index of the polymer increases from 1.58 to 2.08 at 587.6 nm. By adjusting the degree of halogenation through copolymerization or controlling reaction conditions, the refractive index can be carefully tuned. Other essential properties for optical coatings, including thickness and composition uniformity, are studied in detail to demonstrate the practicality of the P4VP-IX (X = I, Br, and Cl) as optical coating materials. P4VP-I_2_ has limited environmental stability, presumably due to CTC dissociation and evolution of I_2_ from the film, while the stability of P4VP-ICl is significantly enhanced due to stronger CTC interaction, suggesting new research directions in the pursuit of HRIPs.

## Supporting Information Available

Schematic of the iCVD system, optical property characterizations of P4VP-IBr and P4VP-ICl, FTIR analysis of P4VP-I_2_ (CTC formation and thermal stability as a function time), and area maps of P4VP, P4VP-IBr, and P4VP-ICl film properties (PDF)
