# Supplementary material for: High Refractive Index Polymer Thin Films by Charge-Transfer Complexation
Source: Macromolecules. 2023 Mar 3;56(5):2113–22. doi: 10.1021/acs.macromol.2c02532 (PMC10019454; doi:10.1021/acs.macromol.2c02532)
Supplement: Supplementary file 1 — ma2c02532_si_001.pdf [file ma2c02532_si_001.pdf]

Supporting information

High Refractive Index Polymer Thin Films by Charge Transfer Complexation

Ni Huo, Wyatt E. Tenhaeff*

Department of Chemical Engineering, University of Rochester, Rochester, NY, 14620

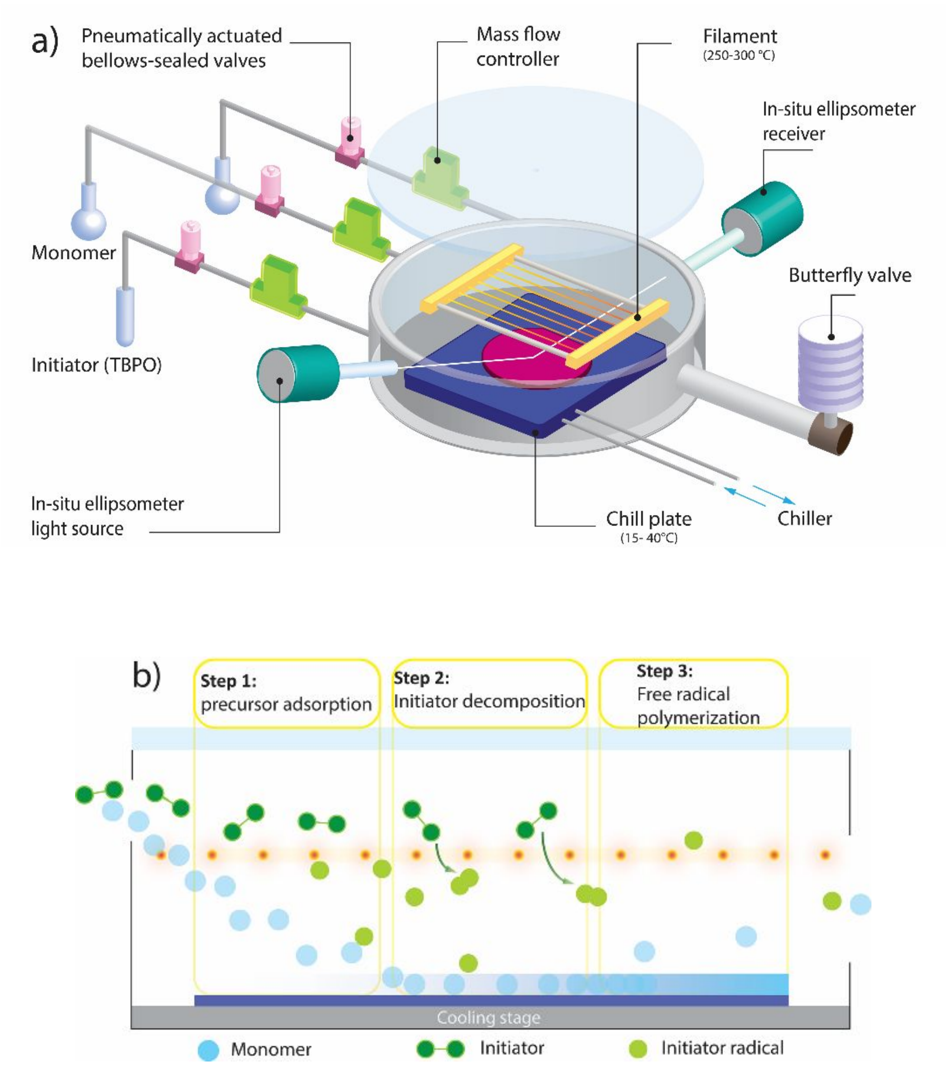

Figure S1. (a) Schematic of initiated chemical vapor deposition (iCVD) system, and (b) conceptual depiction of reaction scheme in iCVD.

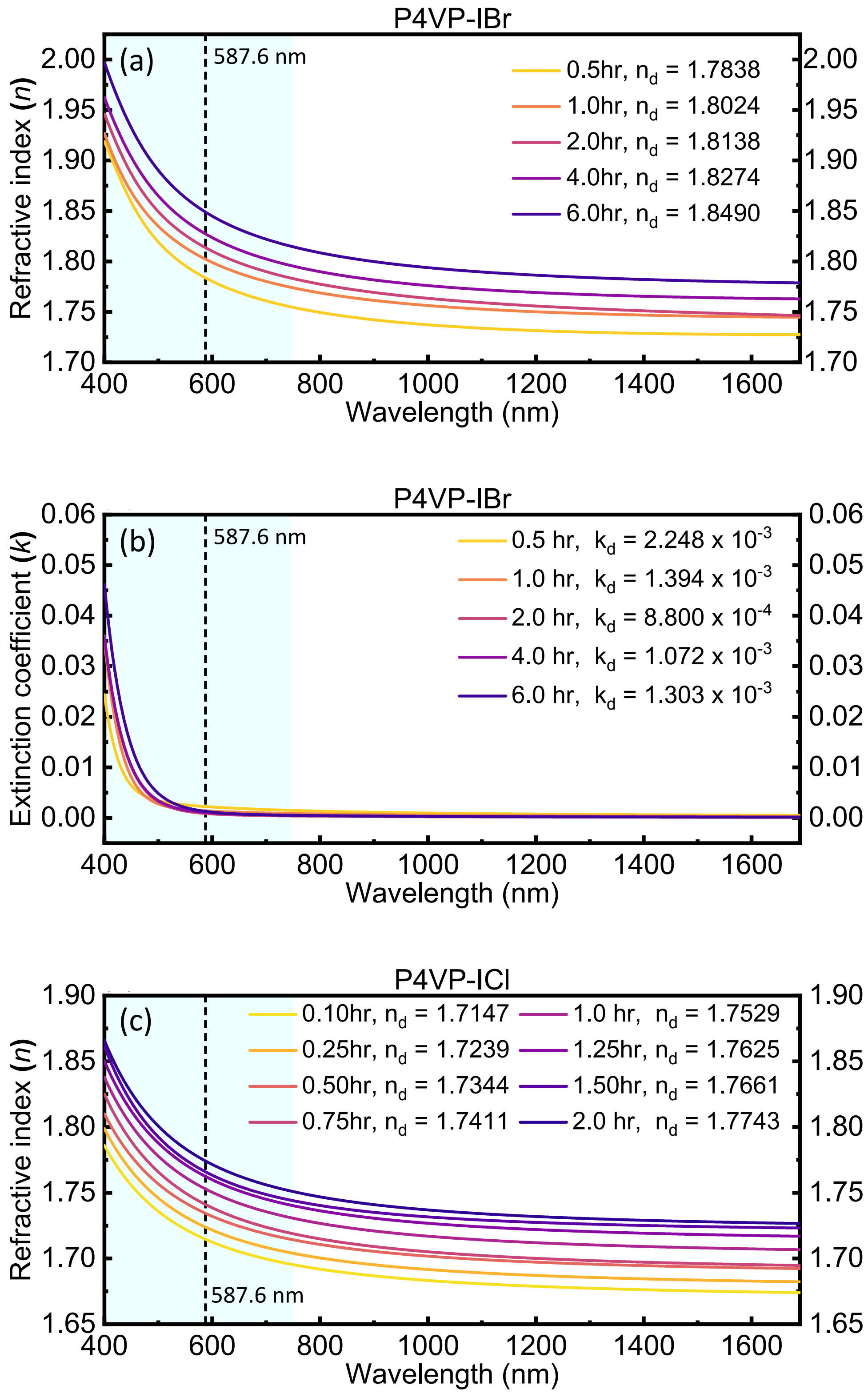
P4VP-IBr
(a)
587.6 nm
0.5hr, n_d = 1.7838
1.0hr, n_d = 1.8024
2.0hr, n_d = 1.8138
4.0hr, n_d = 1.8274
6.0hr, n_d = 1.8490
Refractive index (n)
Wavelength (nm)
P4VP-IBr
(b)
587.6 nm
0.5 hr, k_d = 2.248 x 10^-3
1.0 hr, k_d = 1.394 x 10^-3
2.0 hr, k_d = 8.800 x 10^-4
4.0 hr, k_d = 1.072 x 10^-3
6.0 hr, k_d = 1.303 x 10^-3
Extinction coefficient (k)
Wavelength (nm)
P4VP-ICl
(c)
0.10hr, n_d = 1.7147
0.25hr, n_d = 1.7239
0.50hr, n_d = 1.7344
0.75hr, n_d = 1.7411
1.0 hr, n_d = 1.7529
1.25hr, n_d = 1.7625
1.50hr, n_d = 1.7661
2.0 hr, n_d = 1.7743
587.6 nm
Refractive index (n)
Wavelength (nm)

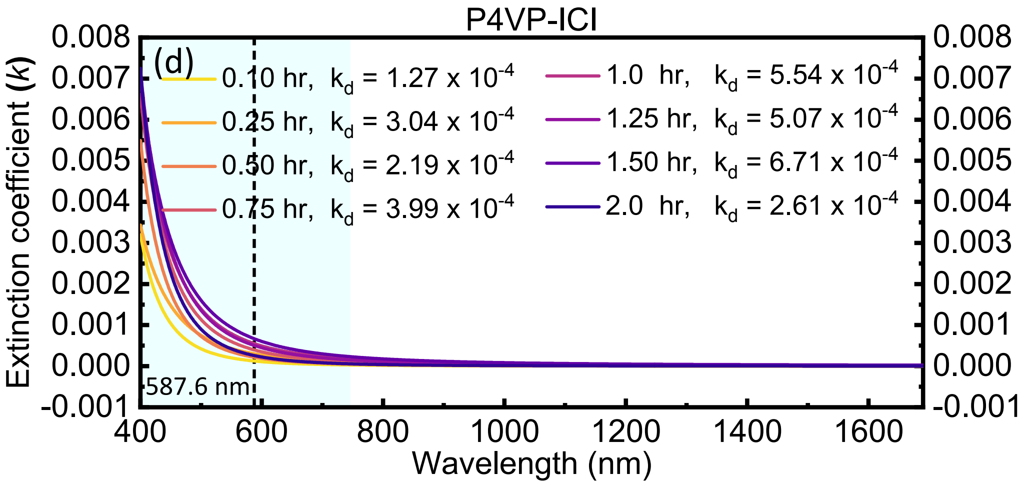

Figure S2. (a) RI spectra of P4VP - IBr; (b): k spectra of P4VP - IBr; (c) RI spectra of P4VP – ICl; (d): k spectra of P4VP – ICl

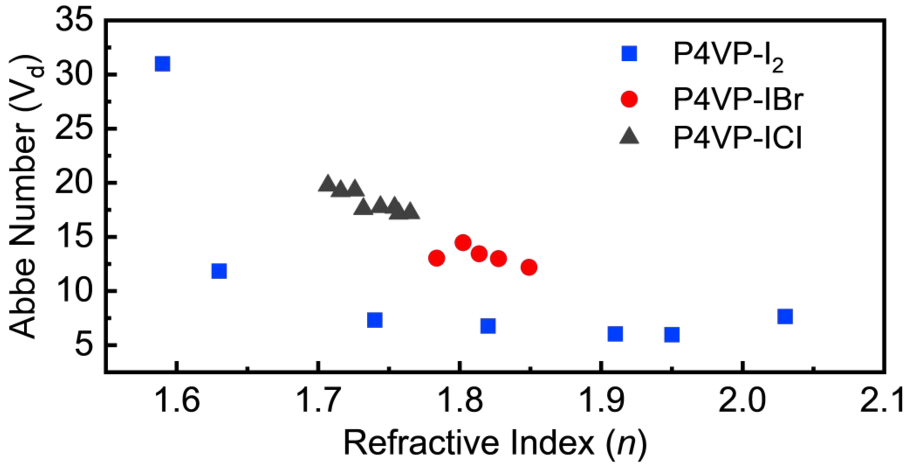

Figure S3. Abbe number of P4VP-IX (X=I, IBr, ICI) plotted against refractive index.

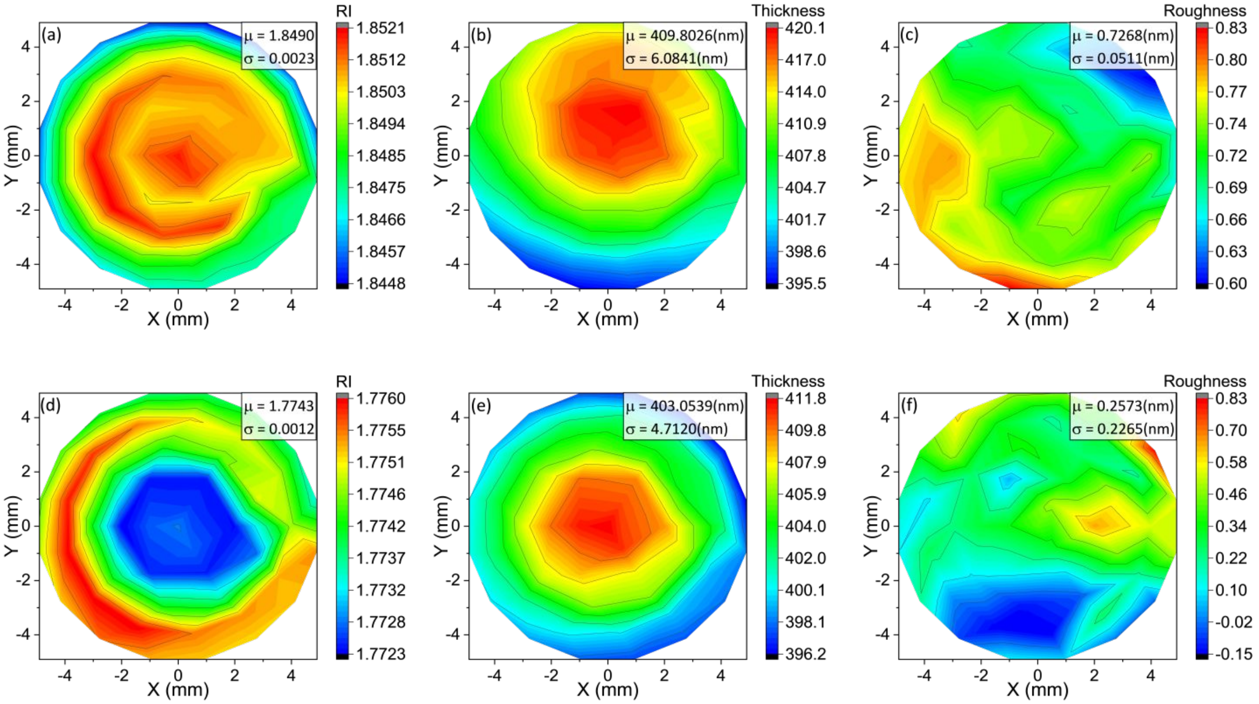

Figure S4. Maps of (a) refractive index, (b) thickness, and (c) roughness of P4VP-IBr thin film coated on Si wafer. Maps of (d) refractive index, (e) thickness, and (f) roughness of a P4VP-ICl thin film coated on Si wafer.

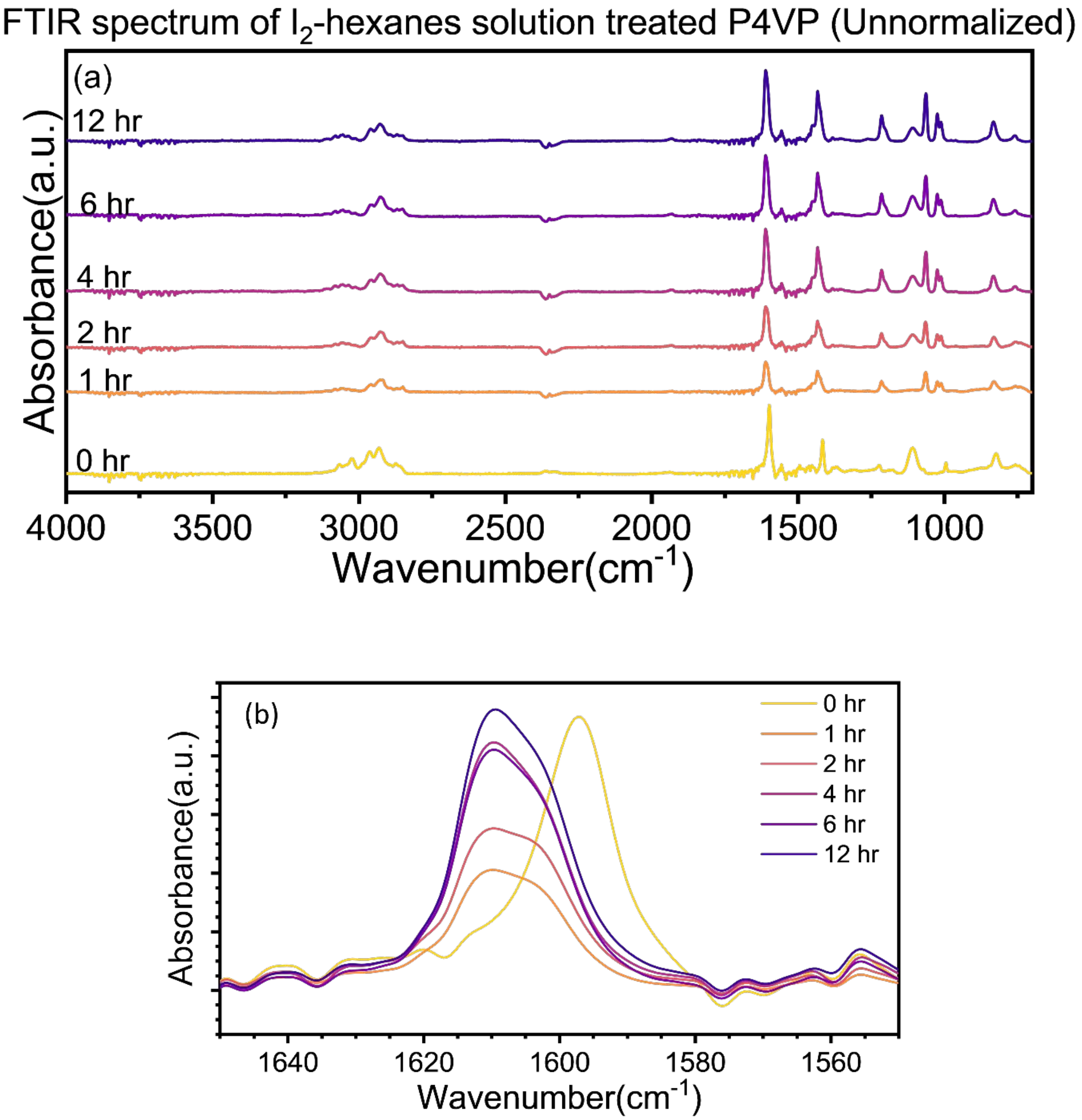
FTIR spectrum of I2-hexanes solution treated P4VP (Unnormalized)
(a)
12 hr
6 hr
4 hr
2 hr
1 hr
0 hr
Absorbance(a.u.)
4000
3500
3000
2500
2000
1500
1000
Wavenumber(cm-1)
(b)
0 hr
1 hr
2 hr
4 hr
6 hr
12 hr
Absorbance(a.u.)
1640
1620
1600
1580
1560
Wavenumber(cm-1)

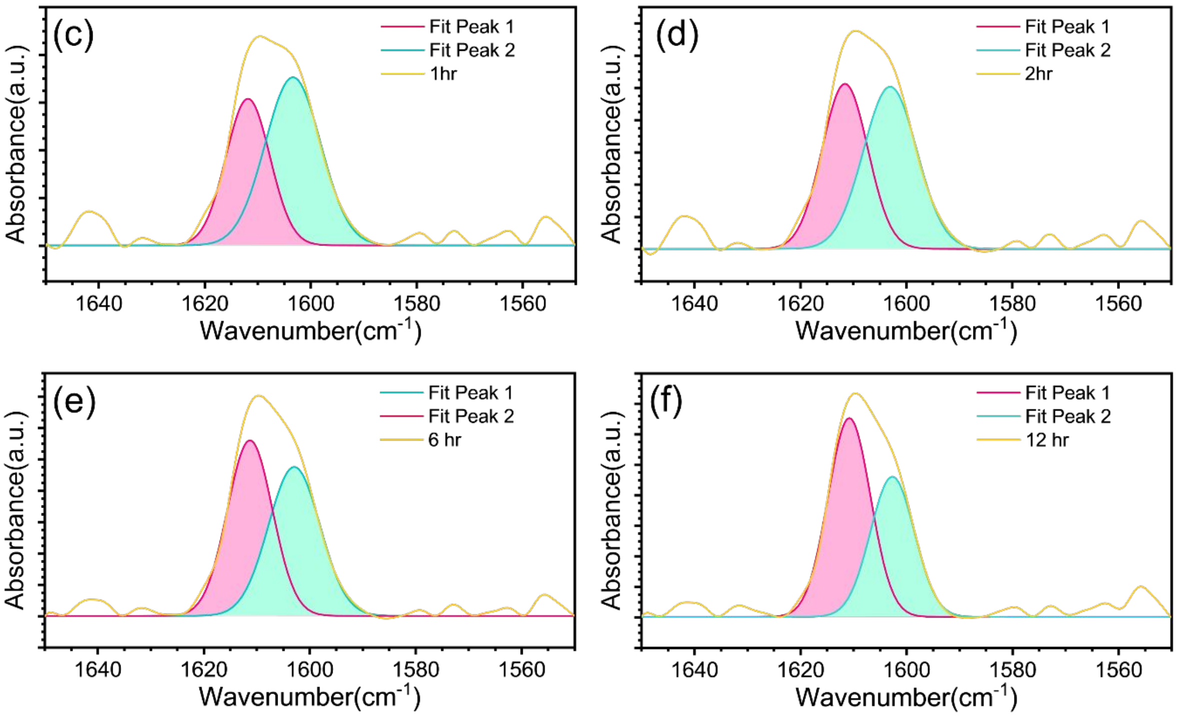

Figure S5. (a) Fourier Transform Infrared (FTIR) spectra of P4VP-I_2 complex thin films with varying I_2-hexanes treatment time, and (b) Close-up view of the fingerprint region (1650-1550 cm^{-1}) in the FTIR spectra. Deconvolution of the peak centered at 1605 cm^{-1} in FTIR spectra of samples with varying treatment time of (c)1hr, (d)2hr, (e)6hr, and(f)12hr. The peak is separated into two peaks: 1603 and 1609 cm^{-1}, indicating the vibration of the pyridine ring and the pyridine ring impacted by electron redistribution due to the formation of the CTC. The integral data of the peaks are presented in Table 1.

Table S1. Peak integrals and integral ratios of two deconvoluted FTIR peaks at 1603 and 1609 cm-1 for sample with varying treatment time.

**Treatment Time (hr)**	**Peak Integral**		**Ratio**
	**1609 cm^{-1}**	**1603 cm^{-1}**	
**1**	0.32	0.46	0.69
**2**	0.41	0.47	0.87
**6**	0.59	0.58	1.02
**12**	0.66	0.47	1.40

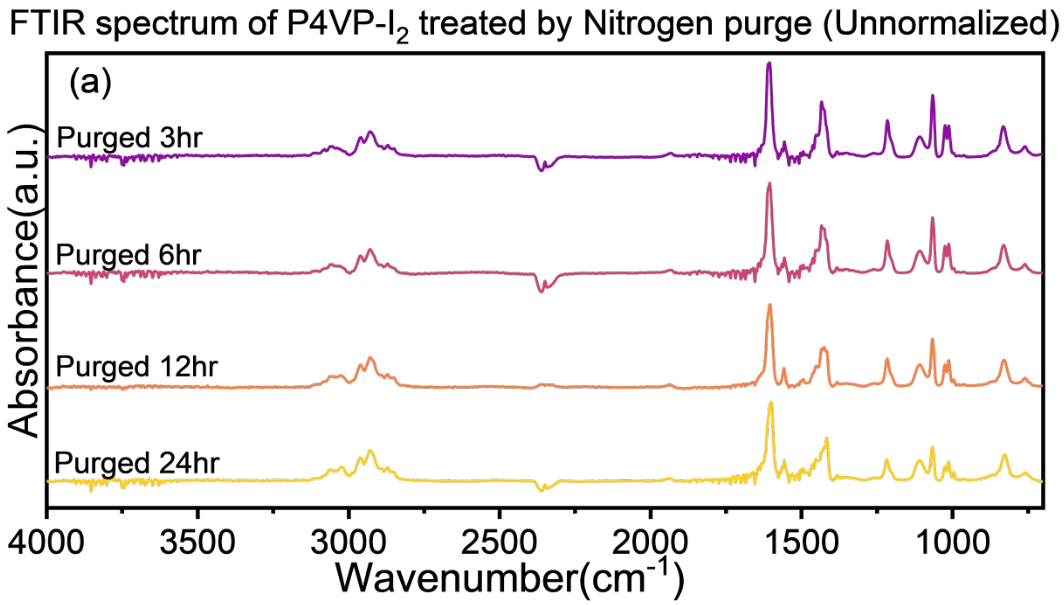
FTIR spectrum of P4VP-I2 treated by Nitrogen purge (Unnormalized)
(a)
Purged 3hr
Purged 6hr
Purged 12hr
Purged 24hr
Absorbance(a.u.)
4000
3500
3000
2500
2000
1500
1000
Wavenumber(cm-1)

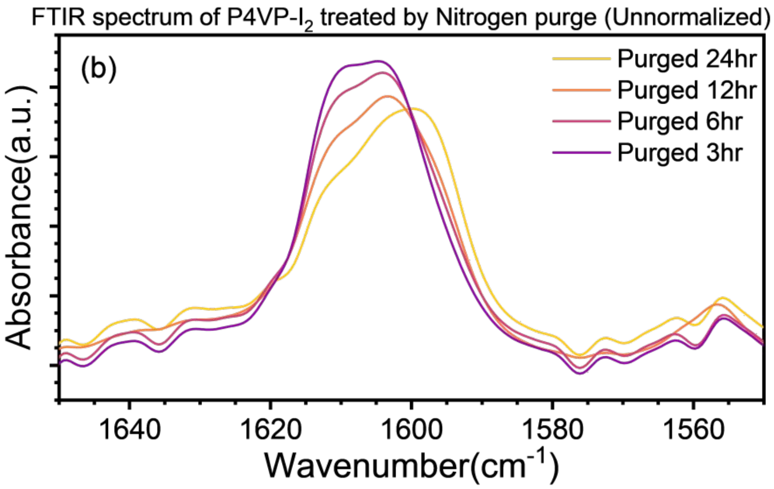
FTIR spectrum of P4VP-I2 treated by Nitrogen purge (Unnormalized)
(b)
Purged 24hr
Purged 12hr
Purged 6hr
Purged 3hr
Absorbance(a.u.)
1640
1620
1600
1580
1560
Wavenumber(cm-1)

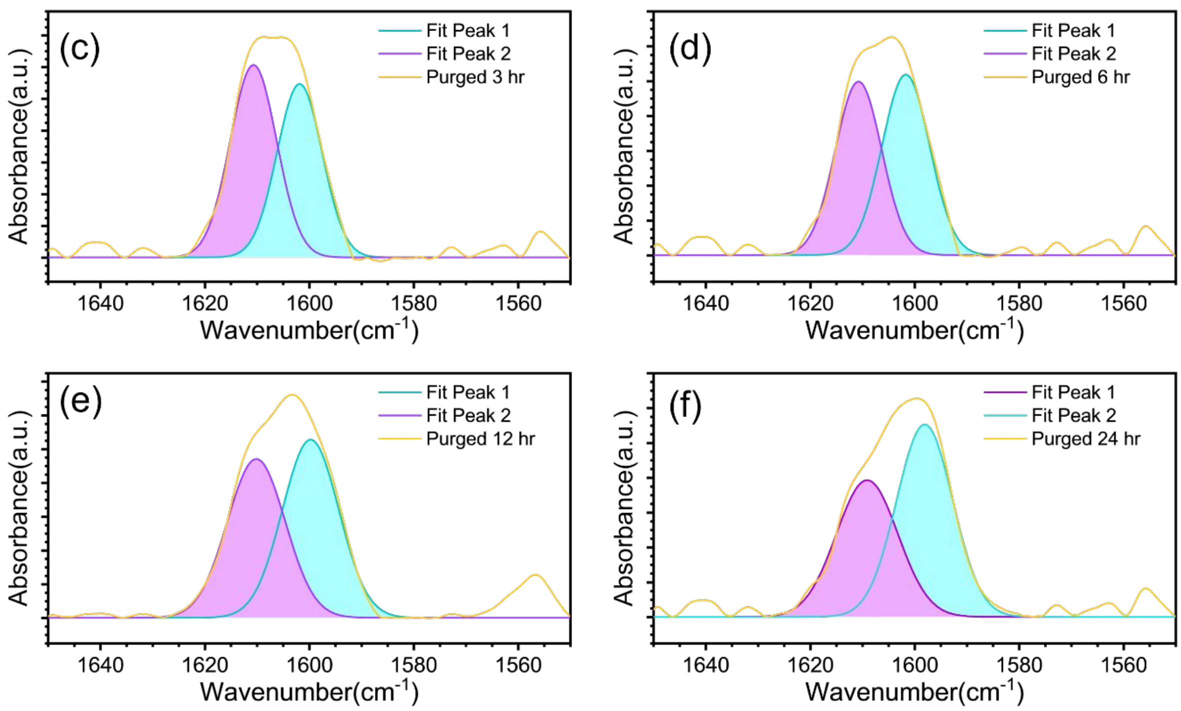

Figure S6 (a) Fourier Transform Infrared (FTIR) spectra of P4VP-I_2 complex thin films with varying nitrogen purging time, and (b) Close-up view of the fingerprint region (1650-1550 cm^{-1}) in the FTIR spectra. Deconvolution of the peak centered at 1605 cm^{-1} in FTIR spectra of samples with varying purge time of (c) 3hr, (d) 6hr, (e) 12hr, and(f) 24hr. The peak is separated into two peaks: 1603 and 1609 cm^{-1}, indicating the vibration of the pyridine ring and the pyridine ring impacted by electron redistribution due to the formation of the CTC. The integral data of the peaks are presented in Table 2.

Table S2. Peak integrals and integral ratios of two deconvoluted FTIR peaks at 1603 and 1609 cm-1 for sample with varying treatment time.

**Purge time(hour)**	**Peak Integral**		**Ratio**
	**1609 cm^{-1}**	**1603 cm^{-1}**	
**3**	0.67	0.59	1.14
**6**	0.55	0.59	0.93
**12**	0.64	0.71	0.90
**24**	0.58	0.74	0.78

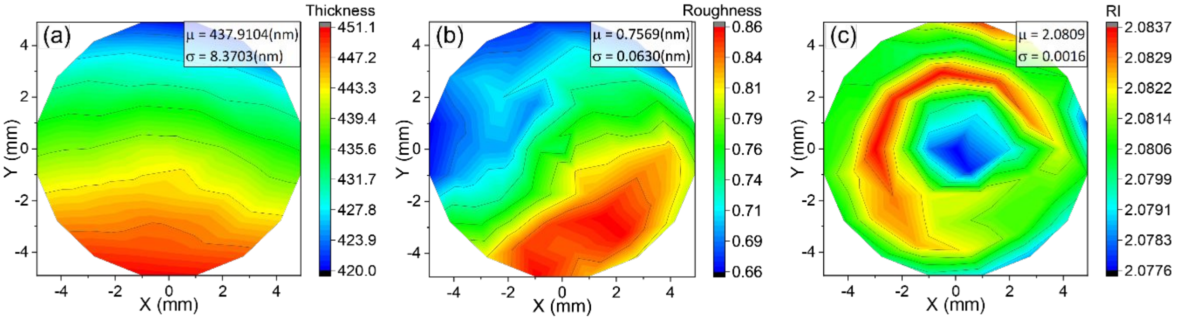

Figure S7. Map of (a) refractive index, (b) thickness, and (c) roughness of a P4VP thin film coated on Si wafer.
